# Phase Evolution and Thermodynamics of Cubic Li_6.25_Al_0.25_La_3_Zr_2_O_12_ Studied by High-Temperature X-ray Diffraction

**DOI:** 10.1021/acs.inorgchem.4c03738

**Published:** 2025-03-18

**Authors:** Øystein Gullbrekken, Kristoffer Eggestad, Maria Tsoutsouva, Benjamin A. D. Williamson, Daniel Rettenwander, Mari-Ann Einarsrud, Sverre M. Selbach

**Affiliations:** Department of Materials Science and Engineering, Norwegian University of Science and Technology, NTNU, Trondheim N-7491, Norway

## Abstract

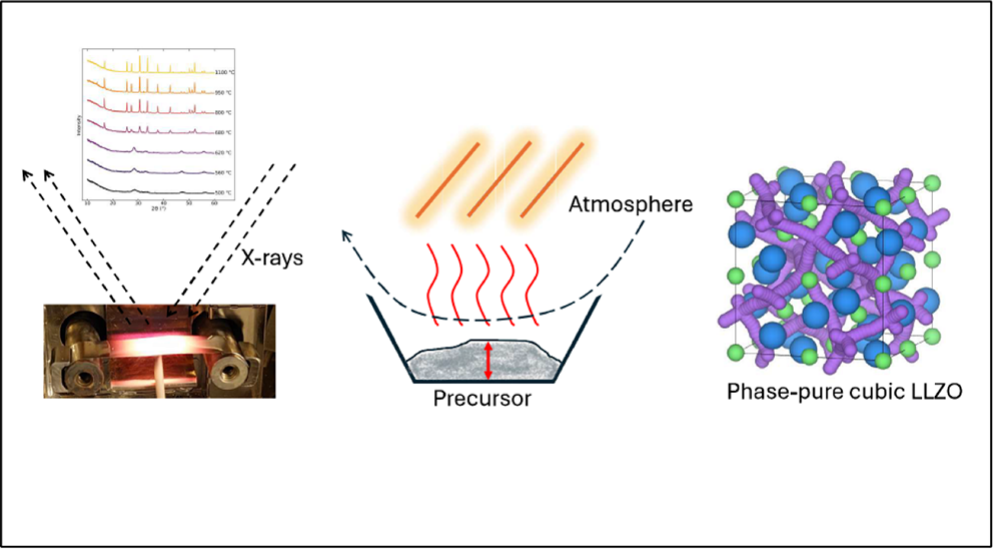

The cubic garnet Li_7_La_3_Zr_2_O_12_ (LLZO) is a prototypical ceramic electrolyte for solid-state Li-ion batteries. While the electrochemical performance of LLZO is well studied, the thermodynamics of the formation of LLZO is not fully understood, and reliable synthesis of phase-pure cubic LLZO requires such knowledge. Here, we report a high-temperature X-ray diffraction (HTXRD) study of the crystallization of Al-doped LLZO from an amorphous gel with different amounts of excess Li. Based on the phases identified in the precursor powders before and during heating, a net chemical reaction for the formation of LLZO is proposed, and its thermodynamic properties are calculated. The sample thickness, and hence the surface exposure to the atmosphere during calcination, strongly affects the phase evolution of cubic LLZO. The configurational entropy of cubic LLZO is estimated to be large and necessary to stabilize cubic LLZO.

## Abstract 2

Here, we report a high-temperature X-ray diffraction (HTXRD) study of the crystallization of Al-doped LLZO from an amorphous gel with different amounts of excess Li. Based on the phases identified in the precursor powders before and during heating, a net chemical reaction for the formation of LLZO is proposed, and its thermodynamic properties are calculated.

## Introduction

Conventional Li-ion battery technology based on liquid electrolytes is reaching its limits with respect to energy density without compromising safety. Batteries with higher energy densities are required to accelerate the reduction of CO_2_ emissions. Solid-state ceramic electrolytes are inherently safer than volatile and flammable liquid electrolytes and can potentially enable batteries with higher energy density. The garnet Li_7_La_3_Zr_2_O_12_ (LLZO) is a promising solid-state electrolyte for next-generation Li-ion batteries due to its high Li^+^ ionic conductivity, nonflammability, and high electrochemical stability against Li anodes and high-voltage cathodes.^1,2^

LLZO is typically synthesized via solid-state reaction or sol–gel synthesis routes.^3−16^ The synthesis of phase-pure LLZO is challenging due to the easy formation of secondary phases, such as the pyrochlore La_2_Zr_2_O_7_ and carbonates, which are detrimental to the performance of an LLZO electrolyte. Furthermore, to prepare high-performance LLZO electrolytes, the fast ion-conducting cubic phase must be stabilized over the tetragonal phase at ambient temperature.

The synthesis method and conditions, such as temperature, atmosphere, excess Li, and type of precursors, govern the evolution of LLZO and potential competing phases, such as pyrochlore and carbonates. High-temperature calcination is necessary to stabilize the cubic phase of LLZO, but it causes evaporation and loss of Li, which might lead to the decomposition of LLZO and the formation of La_2_Zr_2_O_7_.^17,18^ As a consequence, excess Li is normally added to compensate for the Li loss^19−24^ and the excess amount is expected to affect the phase evolution of LLZO. The importance of the atmosphere during the synthesis has been reported in several studies.^17,20,25^ Sol-gel synthesis may involve organic precursors or complexing agents, which will form CO_2_ upon calcination, and the partial pressure of CO_2_ at the surface of the resulting powder has been shown to influence the equilibrium conditions of LLZO formation.^25^

The formation of cubic and tetragonal polymorphs of LLZO competes during high-temperature calcination and subsequent cooling.^26^ Li^+^ vacancies are believed to stabilize the cubic phase, and these are normally obtained by hypervalent doping, e.g., by substitution of Li^+^ with Al^3+^ or Ga^3+^ which creates two Li^+^ vacancies per Al/Ga.^27,28^ Substitution of Zr^4+^ is also possible, for example, with Nb^5+^ or Ta^5+^.^29−32^ Too much excess Li can lead to the stabilization of the tetragonal phase with respect to the cubic phase due to the suppression of Li^+^ vacancy formation.^22,33^ The calcination temperature and time determine the amount of Li loss, concentration of Li^+^ vacancies, and the resulting stability of the cubic versus tetragonal phase. Paolella et al.^25^ showed it is possible to stabilize the cubic LLZO phase without dopants by slowing down the decomposition of lithium carbonate using a stagnant N_2_ atmosphere, and hence increasing the amount of lithium available for reaction.

Even though some methods for stabilizing the cubic phase of LLZO are established, an understanding of the thermodynamics of the LLZO formation and the role of the synthesis conditions is lacking. Here, we report a high-temperature X-ray diffraction (HTXRD) study of the crystallization of an Al-doped LLZO gel. The influence of the initial Li excess and atmospheric exposure during calcination is investigated. We propose a net chemical reaction based on the identified reactants and products and calculate the enthalpy, entropy, and Gibbs energy of this reaction. Finally, we discuss the significance of the highly disordered Li^+^ sublattice for the entropy stabilization of cubic LLZO.

## Methods

### Materials Synthesis

Precursors for the Al-doped LLZO powders (Li_6.25_Al_0.25_La_3_Zr_2_O_12_) were prepared by using an aqueous Pechini-based method.^6^ Precursor solutions of La(NO_3_)_3_·6H_2_O (99.9%, Alfa-Aesar), ZrO(NO_3_)_2_·xH_2_O (99%, Sigma-Aldrich), and Al(NO_3_)_3_·9H_2_O (98%, Sigma-Aldrich), respectively, were made by dissolving the powders in deionized water. The nitrate solutions were standardized thermogravimetrically to determine their exact cation concentrations. The concentrations were 0.2422 ± 0.0004 mol L^–1^ Zr^4+^, 0.1575 ± 0.0009 mol L^–1^ Al^3+^, and 0.3226 ± 0.0001 mol L^–1^ La^3+^. Stoichiometric amounts of the solutions were added to a glass beaker, along with LiNO^3^ (99.99%, Sigma-Aldrich) powder that had been dried overnight at 110 °C. Different amounts of LiNO_3_ were added, corresponding to 0, 10, and 20 mol % excess lithium. Anhydrous citric acid (99.5%, Sigma-Aldrich) and ethylene glycol (99.8%, Sigma-Aldrich) were then added as complexing and polymerizing agents, respectively. The amounts of citric acid and ethylene glycol added were double the total molar content of all cations. The solution was heated to 100 °C on a hot plate under vigorous stirring, and after 1–2 h, the solution had turned into a white foam, which was heated in a furnace at 250 °C for 1 or 2 h. The resulting brown resin was removed from the glass beaker and crushed into a coarse powder with a mortar. This powder was calcined in an alumina crucible at 500 °C for 6 h, with heating and cooling rates of 200 °Ch^–1^, to produce a black precursor powder. The resulting precursor powder was used as the starting material for the high-temperature X-ray diffraction studies and for calcination at higher temperatures in a furnace to study the phase evolution to cubic LLZO. Powders calcined at a relatively modest temperature of 500 °C are not expected to be significantly contaminated by Al from alumina crucibles, which is known to occur at higher temperatures.^34^ Precursor powders containing 0, 10, and 20% excess Li were calcined for 6 h at 850 °C, 900 °C, and 1000 °C, respectively.

### Materials Characterization

Room temperature X-ray diffraction (XRD) was conducted on a Bruker D8 Focus instrument with Cu–K_α_ radiation of wavelength λ = 1.5418 Å from 2θ = 10° to 80°, using a step size of 0.014° and a dwell time of 1 s.

High-temperature X-ray diffraction (HTXRD) studies were conducted with a Bruker D8 Advance using Cu–K_α_ radiation in the 2θ range of 10° to 80°, with a step size of 0.016° and a step time of 1 s. Powders milled in isopropanol with 5 mm diameter zirconia balls and dried in a rotary evaporator were dispersed in ethanol and manually deposited as a thin film covering a Pt strip used for heating the sample. The sample with 10% excess Li was analyzed twice, with normal (similar to the other samples) and thinner deposition thickness. The sample chamber was sealed and continuously flushed with synthetic air during the experiments. The samples were heated from 500 °C to 1000 °C (1100 °C for the 20% excess Li sample) in steps of 20 °C between 500 °C and 700 °C, and in steps of 50 °C from 700 °C to 1000/1100 °C. The heating rate was set to 0.2{°Cs^–1^, and the temperature was held at each specified temperature for 2 h before further heating, including data collection. A final XRD pattern was recorded after cooling to 30 °C. Data collection started immediately after reaching each target temperature. The lattice parameters and crystallite sizes of the LLZO and La_2_Zr_2_O_7_ phases were determined by Pawley fitting using TOPAS software. The background, described by a Chebyshev polynomial with 10 terms, along with the lattice parameter and crystallite size, were refined. The sample displacement was refined but constrained to scale linearly with temperature. Note that lattice strain was not refined due to the limited available 2θ window and resulting uncertainty in the deconvoluting strain and crystallite size.

Mass loss during calcination of the precursor powders was investigated by thermogravimetry (TG) using alumina crucibles. The powder samples (∼50 mg) were heated from room temperature to 1200 °C with a heating rate of 3 °Cs^–1^, followed by an isothermal step at 1200 °C for 1h. The sample chamber was flushed with 20 mL/min of synthetic air during the analysis. The evolution of gaseous species during TG analysis was analyzed by mass spectrometry (MS). The combined TG-MS experiments were conducted in a Netzsch STA449 C Jupiter TG, in the differential thermal analysis setup, connected to a QMS 403 C Aëolos mass spectrometer. The mass spectrometer was set up to detect species with atomic masses between 1 and 100.

The morphology of the powders after the HTXRD experiments was characterized by scanning electron microscopy (SEM, FEI Apreo).

### Computational Details

La_2_O_2_CO_3_ (space group: *C*2/*c*) was investigated using density functional theory (DFT) calculations with the Vienna ab initio Simulation Package (VASP)^35−37^ to determine its heat capacity. Lattice parameters and atomic positions for the 48-atom primitive cell were optimized using the PBEsol functional^38^ with a 5 × 5 × 2 Γ-centered *k*-point mesh and a plane wave cutoff energy of 600 eV. The projector-augmented wave method^39^ (PAW) was used to describe interactions between cores and valence electrons (La: (5s^2^, 5p^6^, 5d^1^, 6s^2^), C: (2s^2^, 2p^2^), and O: (2s^2^, 2p^4^)). The relaxed structure was obtained with convergence criteria of 10^–4^ eVÅ^–1^ and 10^–8^ eV for the forces on all ions and the electronic ground state, respectively. Relaxed structure parameters are given in Tables S1 and S2. The enthalpy of La_2_O_2_CO_3_ was calculated by taking the energy difference between the optimized La_2_O_2_CO_3_ structure and the optimized reference structures for the constituent elements. The vibrational entropy was calculated within the finite-differences method formalized within the Phonopy code^40^ using a 3 × 3 × 2 supercell. The electronic structures of the supercells were optimized using PBEsol and an electronic convergence criterion of 10^–8^ eV and a 3 × 3 × 2 Γ-centered *k*-point grid.

## Results

**Figure 1:**
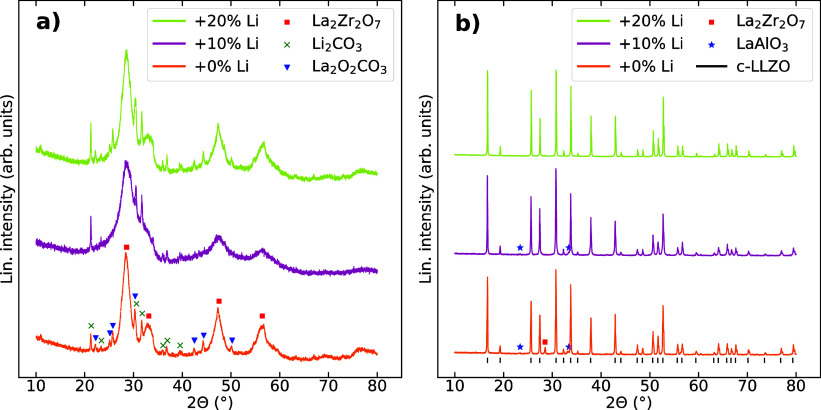
(a) XRD patterns of precursor powders with different excess of Li calcined at 500 °C for 6 h. La_2_Zr_2_O_7_, Li_2_CO_3_, and La_2_O_2_CO_3_ diffraction lines from PDFs no. 01–073–0444, 00–022–1141, and 00–037–0804 are shown, respectively. (b) XRD patterns of powders with 0, 10, and 20% excess Li calcined at different temperatures of 850, 900, and 1000 °C, respectively. The cubic LLZO peaks and LaAlO_3_ peaks are from PDFs nos. 00–063–0174 and 00–073–0268, respectively.

XRD patterns of the precursor powders with different nominal amounts of excess Li are shown in Figure 1a. Bragg reflections assigned to the pyrochlore La_2_Zr_2_O_7_, Li_2_CO_3_, and La_2_O_2_CO_3_ phases are indicated in the XRD pattern of the precursor powder with 0% excess Li. The same reflections can be seen in the diffractogram of the precursor powder with 20% excess Li. The pyrochlore reflections, e.g., at 2θ = 47° and 56° are not as distinct in the diffractogram of the 10% excess Li precursor powder. The pyrochlore reflections are broad in all three XRD patterns, and the peak profiles fitted using the fundamental parameters approach indicate crystallite sizes of 3–5 nm. The other phases display narrower reflections, implying that they are not nanocrystalline. Amorphous phases are also expected to be present from the shape of the diffractogram baselines.

The X-ray diffractograms of the powders after high-temperature calcination are presented in Figure 1b. Some La_2_Zr_2_O_7_ and LaAlO_3_^41^ are present in the powder with 0% excess Li, most likely due to Li loss and deficiency. The other powders are mostly phase-pure, with traces of LaAlO_3_ and La_2_Zr_2_O_7_.

**Figure 2:**
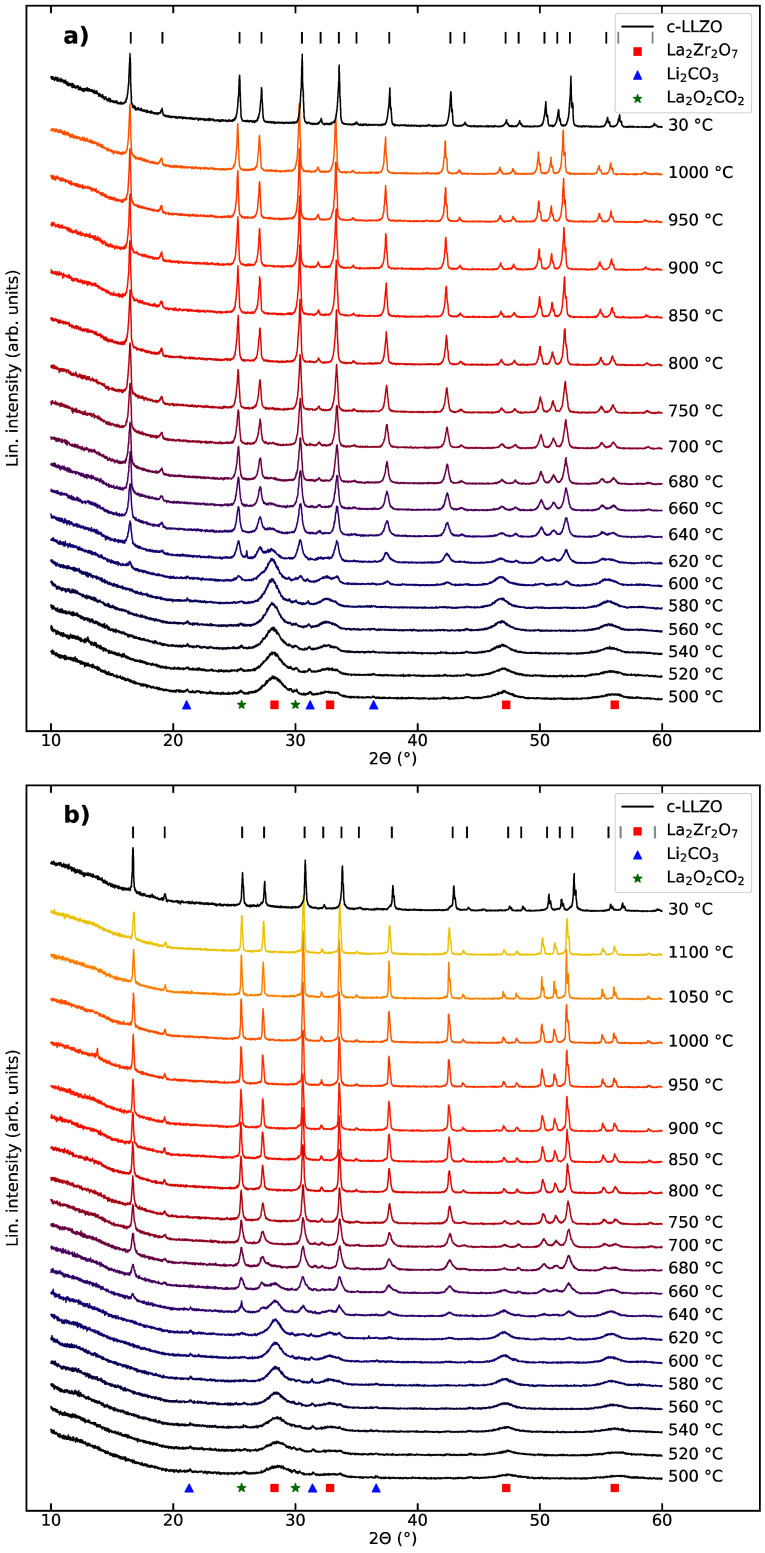
HTXRD patterns of powders with (a) 0% Li excess from 500 °C to 1000 °C and (b) 20% Li excess from 500 °C to 1100 °C. The XRD patterns obtained at 30 °C after the HTXRD are shown at the top of the plots.

The HTXRD patterns of powders with 0 and 20% excess Li are shown in Figure 2. Upon heating from 500 to 600 °C, La_2_Zr_2_O_7_ crystallizes, and the progressively sharper diffraction lines indicate crystallite growth as the temperature increases to 600 °C. The cubic LLZO phase appears at 620 and 640 °C in the powders with 0 and 20% excess Li, respectively. Upon further heating, the La_2_Zr_2_O_7_ phase gradually disappears, and the cubic LLZO phase becomes dominant. The La_2_Zr_2_O_7_ peaks are not visible for temperatures higher than 700 °C. The crystallization of the cubic LLZO phase continues during further heating, and both samples are phase pure after the HTXRD experiments, as shown in the final diffractograms recorded at 30 °C. The diffraction lines of Li_2_CO_3_ and La_2_O_2_CO_3_ phases are not as easily detected in the HTXRD patterns as in the ex situ XRD patterns in Figure 1a.

**Figure 3:**
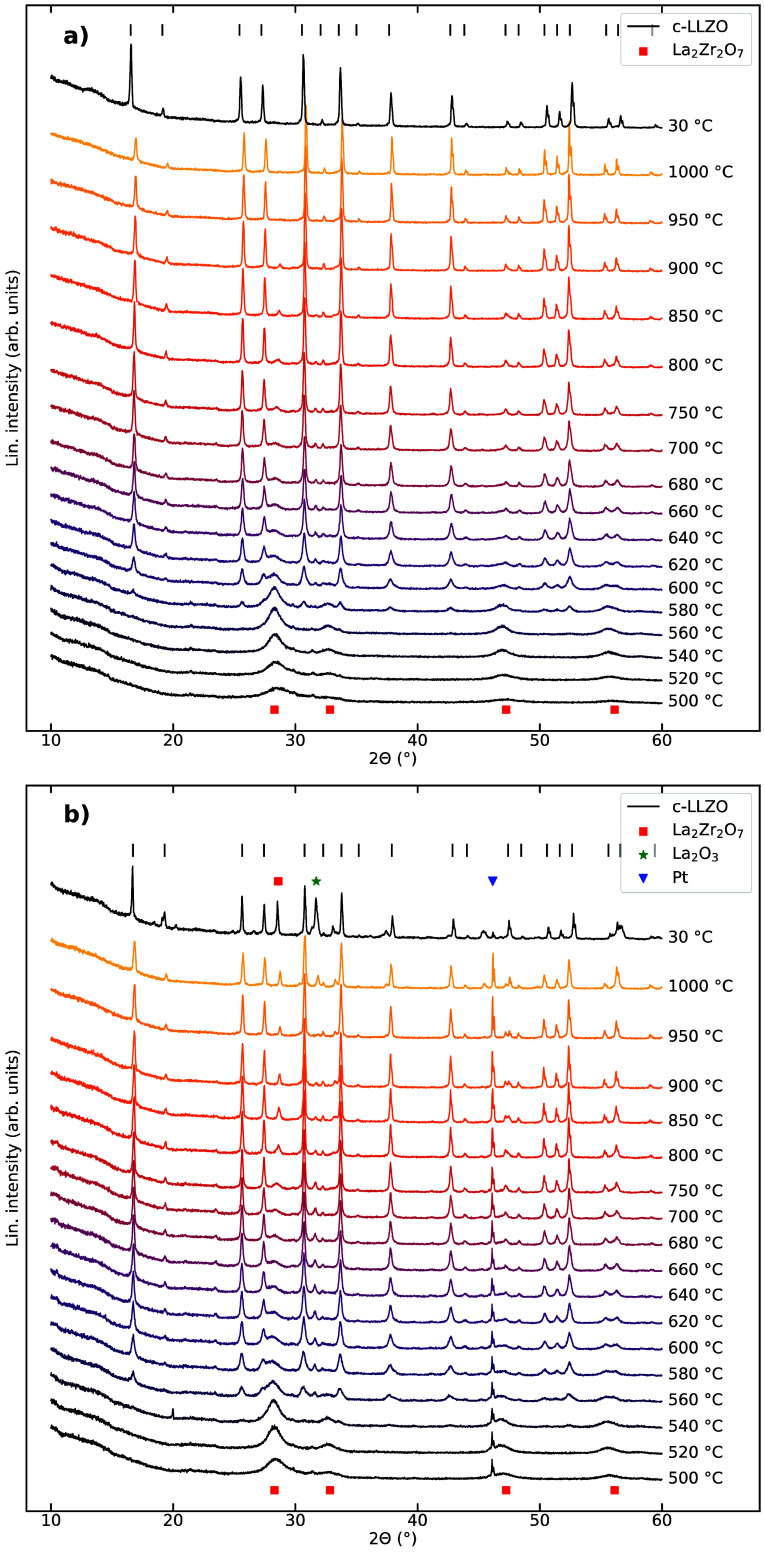
HTXRD patterns of powder with 10% Li excess from 500 °C to 1000 °C. (a) Normal deposition thickness. (b) Deposited as a thin layer on the Pt strip. The green asterisk and red square denote La_2_Zr_2_O_7_ and La_2_O_3_ (PDF no. 04–005–6788) impurity phases, respectively. The blue triangle denotes a Pt peak visible due to the thin deposition. The XRD patterns obtained at 30 °C after the HTXRD are shown at the top of the plots.

Two sets of HTXRD patterns of the sample with 10% excess Li are presented in Figure 3, where two different deposition thicknesses were used (Figure 3a normal thickness, Figure 3b thinner sample) to evaluate the influence of atmospheric exposure to the powder and the relative surface area of the powder. As for the X-ray patterns of the powders with 0 and 20% excess Li, La_2_Zr_2_O_7_ appears as the first crystalline phase. The cubic LLZO phase first appears at 600 °C in the sample with normal deposition thickness (Figure 3a), while it appears at 560 °C in the thin sample. The major pyrochlore diffraction line at 2θ = 28° disappears at 950 °C in the sample with normal deposition thickness, while it remains to the end and even increases in intensity at the highest temperatures for the thin sample. La_2_O_3_ is also present in the thin sample at 1000 °C. The XRD patterns recorded at 30 °C indicate substantial amounts of both La_2_Zr_2_O_7_ and La_2_O_3_ phases present in the thin sample, while the sample with normal thickness is phase pure.

**Figure 4:**
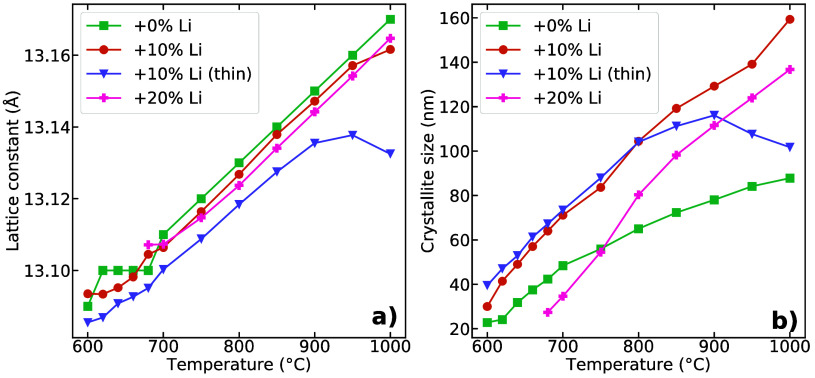
Pawley refined (a) the lattice parameter and (b) crystallite size (LVol-IB) of the cubic LLZO phase upon heating. Uncertainty from the refinement is smaller than the symbol size.

The lattice parameter and crystallite size of the cubic LLZO phase as a function of temperature are shown in Figure 4. The slopes in the linear region of the lattice parameters in Figure 4a are quite similar, implying similar thermal expansion coefficients. Deviations from a linear relation are obvious at lower temperatures, where small amounts of cubic LLZO are present, making the Pawley analysis less certain. The sample with 10% Li excess deposited as a thin layer exhibits a decrease in the lattice above ∼900 °C. The crystallites of cubic LLZO grow as expected with increasing temperature, as seen from Figure 4b. The two samples with 10% Li excess display similar crystallite growth until about 900 °C from where the refined size of cubic LLZO crystallites in the thin sample apparently shrinks, and this contradiction is discussed further below. The LLZO crystallites in the sample with 0% Li excess apparently grow slower than in the other samples.

## Discussion

### Thermodynamics of Formation of LLZO

Based on the identified reactants and products from HTXRD, we propose the following net chemical reaction to take place during the heating of the precursor gel:
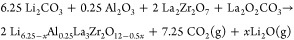
1

**Figure 5:**
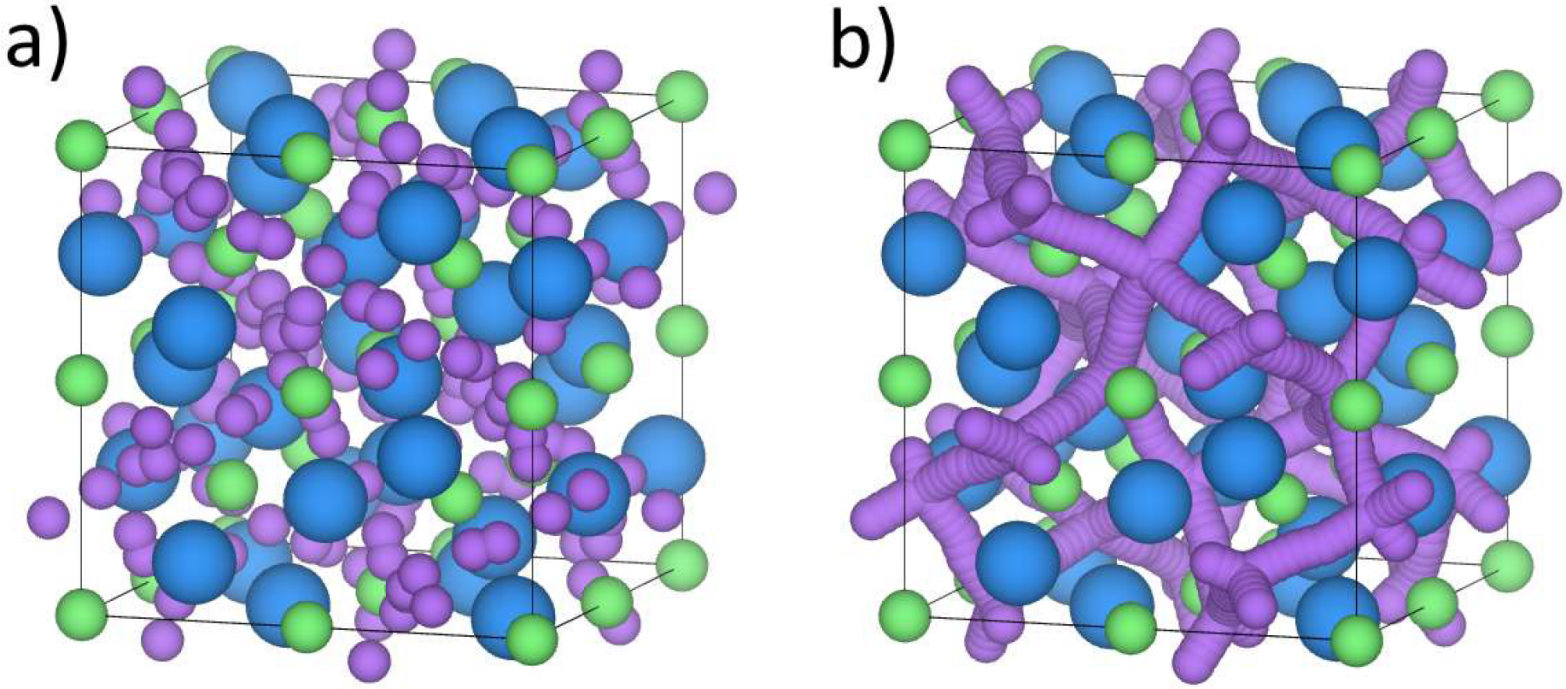
Illustration of cubic LLZO unit cell using VESTA.^49,50^ Li is purple, La is blue, and Zr is green. Oxygen is omitted for clarity. (a) Li positions are displayed. (b) Possible Li pathways are displayed by connecting the nearest Li positions, following ref (51).

**Figure 6:**
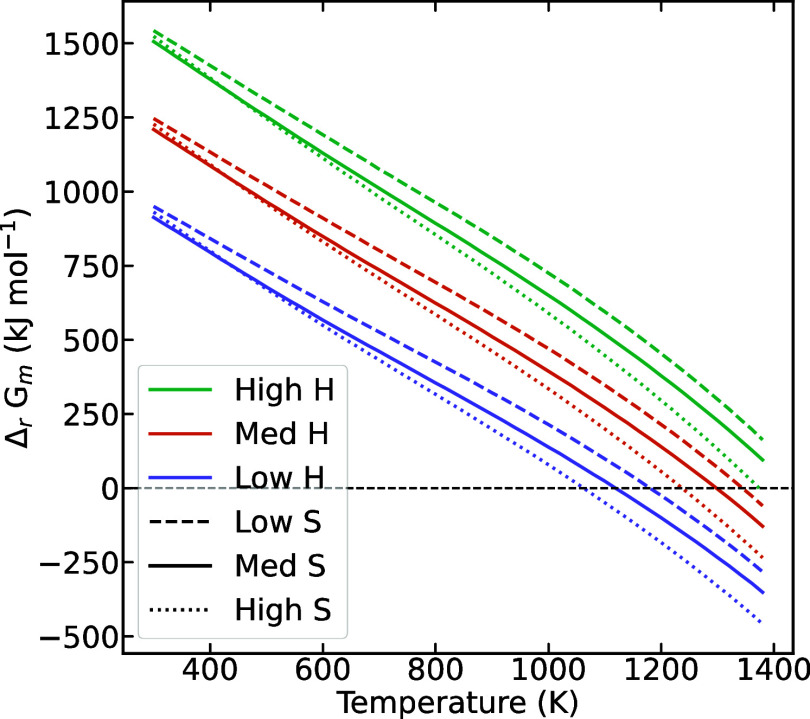
Molar Gibbs energy of reaction (1), employing upper, middle, and lower estimates of the enthalpy and entropy values.

The aluminum is assumed to be present as amorphous alumina after calcination at 500 °C. LLZO is present in the cubic phase, and we assume that Li evaporates as Li_2_O gas.^17^ We have calculated the enthalpy and entropy of reaction (1) using thermodynamic data from literature on Li_2_CO_3_,^42^ Al_2_O_3_,^42^ La_2_Zr_2_O_7_,^43^ tetragonal Li_7_La_3_Zr_2_O_12_,^44^ Li_2_O,^42^ and CO_2_,^42^ and DFT-calculated heat capacity for La_2_O_2_CO_3_. The enthalpy difference between the cubic and tetragonal phases of LLZO is assumed to be relatively small, and Al doping is assumed not to significantly influence the thermodynamic properties of LLZO. However, the Li^+^ sublattice of cubic LLZO is characterized by positions with partial occupancies and is highly disordered compared to the tetragonal phase^28,45^ and could thus contribute to the entropy. The Li^+^ sublattice of cubic LLZO is illustrated in Figure 5. We estimated the configurational entropy of cubic LLZO using the relation *S* = *k* ln *W*, where *k* is the Boltzmann constant and *W* is the number of independent configurations. The number of possible configurations of cubic LLZO was calculated using the primitive unit cell of cubic LLZO, which contains 28 Li, 12 La, 8 Zr, and 48 O atoms. There are 60 positions that Li can occupy, but 24 of them are too close to exist simultaneously, as these are the 96h Wyckoff sites. This gives  2^24^ configurations per primitive unit cell, where *n*_Li,vac_ is the number of Li^+^ vacancies in the unit cell. Additionally, we multiply the number of configurations by four to account for the Al doping, assuming that Al only occupies the tetrahedral positions.^45,46^ Doping with 1 Al per primitive unit cell creates two Li^+^ vacancies. The maximum number of configurations per primitive unit cell is then *W*_max_ =  2^24^ × 4 = 1.706 × 10^16^, which when inserted into *S* = *k* ln *W* gives a configurational entropy of 77.7 J mol^–1^ K^–1^ for cubic LLZO, corresponding to 12.4 J mol^–1^ K^–1^ per Li^+^. For comparison, the configurational entropy of highly disordered α-AgI is 15.0 J mol^–1^ K^–1^ per Ag^+^.^47,48^ Note that Li loss can induce more lithium vacancies, which will create more possible configurations. However, the logarithmic dependence of the number of configurations on the entropy means that the effect on the entropy will be weak. All *W* configurations are assumed to be equally probable, which is obviously not the case regarding the Li^+^ sublattice; hence, the obtained value must be regarded as an upper estimate of the configurational entropy. We calculated lower, middle, and upper estimates of the enthalpy, entropy, and resulting Gibbs energy of the total reaction (1) by scaling all input values by a factor of 0.95, 1, or 1.05, respectively, based on uncertainties in data. The plots for the Gibbs energy of the total reaction (1) are displayed in Figure 6.

The HTXRD patterns in Figures 2 and 3 display the transition from the La_2_Zr_2_O_7_ precursor phase to the cubic LLZO phase. The cubic LLZO phase first appears between 600 °C and 640 °C in the samples deposited with normal thicknesses. There is no clear correlation between the initial amount of excess Li in the samples and the onset and evolution of the cubic LLZO phase. However, there is a relation between the thickness of the deposited sample and the LLZO formation. In the thin sample with 10% Li excess shown in Figure 3b, cubic LLZO appears at a lower temperature of 560 °C compared to the other materials. Additionally, the La_2_Zr_2_O_7_ phase does not fully disappear but instead the amount increases upon heating above 750 °C. As shown in Figure S3b, the crystallite size of the La_2_Zr_2_O_7_ phase increases above 700 °C in the thin sample. Apparently, the phase formation is influenced by the thickness of the sample, which suggests that the surface area, particle size, and oxygen gas exposure are important in understanding LLZO formation. The increase in the amount of the La_2_Zr_2_O_7_ and La_2_O_3_ phases indicates that extensive Li loss has occurred during the heating of the thin sample,^24^ which might be caused by the higher relative surface area of the thin sample.^52^ It is possible that temperature gradients form in the samples during the experiments, but we expect these to be small, considering the generally thin deposition layer. The La_2_Zr_2_O_7_ phase disappears at lower temperatures in the XRD patterns of the samples deposited with normal thickness. Less Li loss in these samples means that more Li is available for LLZO formation at the expense of La_2_Zr_2_O_7_, thus enhancing the formation kinetics. Notably, we observe La_2_Zr_2_O_7_ in the ex situ sample of 0% excess Li (Figure 1b), but not in the in situ HTXRD pattern of the 0% excess Li sample at 850 °C (Figure 2a). We believe this is due to the reaction with the alumina crucible during heating of the ex situ sample, which consumes lithium to form γ-LiAlO_2_ causing lithium deficiency and formation of La_2_Zr_2_O_7_. This reaction does not occur on the Pt strip during the HTXRD experiment; hence, La_2_Zr_2_O_7_ does not form.

There is some discrepancy between the experimental observations and the predictions of the reaction. We observe that cubic LLZO starts to form around 600 °C in the HTXRD experiments, but the Gibbs energy becomes negative around 800 °C for the case with the low estimate of enthalpy and high estimate of entropy, giving the lowest Gibbs energy. There can be several reasons for this discrepancy. Amorphous phases not detectable by XRD can be present, which influence the progress of the reaction. For example, it has been shown that excess Li can lead to the formation of an amorphous/low-crystalline γ-LiAlO_2_ impurity phase, which could also influence the Al concentration in LLZO.^41^ The observation that the sample thickness influences the reaction indicates that atmospheric exposure plays a role. It is possible that some partial reaction steps require oxygen gas to be present, which is not captured by the proposed reaction (1). The partial pressure of oxygen at the surface could thus influence both the reaction kinetics and thermodynamics. In the same way, the partial pressure of CO_2_ near the powder surface likely influences the reaction. If carbonaceous material is present in the precursor powders, this can consume oxygen, and the exothermic combustion reaction can create local hotspots, which accelerate the reaction (see Figure S2). The sample chamber was continuously flushed by synthetic air during the HTXRD experiments, and consequently, the thickness of the powder layer and surface exposure to the atmosphere will affect the reaction progress. A study by Larraz et al.^53^ found that a low-temperature cubic phase of hydrated LLZO is stable at temperatures as low as 200 °C by the insertion of water molecules into the garnet structure and H^+^/Li^+^ exchange. The TG-MS experiments showed the release of water from the precursor powder between 200 °C and 500 °C, but also between 600 °C and 800 °C (see Figure S1), which suggests that some H^+^ might even come from the garnet structure. The thermal decomposition of the potential phase boehmite (AlO(OH)) would also lead to dehydroxylation and formation of water.^54^ The cubic structure observed at temperatures around 600 °C could potentially be a hydrated cubic LLZO. Experimental uncertainties such as a potential temperature gradient in the sedimented layer and local variations of the layer can also explain the above discrepancy.

The thermal expansion coefficient (TEC) calculated from the slope of the lattice parameter with respect to temperature (Figure 4a) varied from 13.5 to 16.0 × 10^–6^ K^–1^ (700–900 °C), which corresponds well with literature values.^55^ The TECs are presented in Table 1. There does not seem to be a relation between the TEC and excess Li content in the samples. Notably, the lattice parameter of the sample with 10% excess Li (thin) decreased at the highest temperatures investigated. A possible explanation for this behavior is Li loss, as Zhan et al. showed that a lower content of Li in cubic Al-doped LLZO caused a reduction of the lattice parameter.^56^ The crystallite size of cubic LLZO, shown in Figure 4b, in the sample with 10% excess Li deposited as a thin layer apparently starts to decrease above 900 °C. This apparent reduction in crystallite size may stem from two sources: first, Li loss and concomitant vacancies may cause microstrain in the lattice, which our refinement misattributes as size broadening of the Bragg peaks. Second, the apparent reduction in crystallite size coincides well with the increase in the amount of the La_2_Zr_2_O_7_ phase at high temperatures in the HTXRD patterns displayed in Figure 3b, and could thus indicate LLZO decomposition to form La_2_Zr_2_O_7_ and La_2_O_3_ due to the Li loss.

**Table 1:** Thermal Expansion Coefficients (TECs) Calculated from Lattice Parameters as a Function of Temperature in Figure 4a

Sample	TEC (× 10^–6^K^–1^)
0% Li	16.0
10% Li	15.4
10% Li (thin)	13.5
20% Li	14.5

### Pechini Synthesis to Prepare Precursor Powders

The XRD patterns of the precursor powders after calcination to 500 °C for 6 h show that three phases are present: La_2_Zr_2_O_7_, Li_2_CO_3_, and La_2_O_2_CO_3_. The acid/base properties of the cations will influence their complexation with citric acid. By considering the charge density of the cations and their ability to bond to the acid group of citric acid, Li^+^ can be viewed as a base, La^3+^ as a weak base or amphoteric, and Al^3+^ and Zr^4+^ as acidic. We expect that Li^+^ and La^3+^ form weaker complexes with citric acid than the two other cations, and this could result in an inhomogeneous distribution of these cations and segregation in the resulting gel.^57^ Potentially, this could influence the kinetics of the high-temperature calcination to form the cubic LLZO. Because Li^+^ and La^3+^ are more basic, they are also more likely to form carbonates, as commonly observed for Pechini-type synthesis routes.^57^

Upon calcination of the gel, nitrates are decomposed first, while at higher temperatures, carbonates will be formed, i.e., Li_2_CO_3_ and La_2_O_2_CO_3_, due to the decomposition of organics. The black color of the precursor powders indicates the formation of carbonaceous compounds after partial decomposition of the polymer. The TG-MS analysis of the precursor powder, shown in Figure S1, displays the emission of carbon dioxide from 200 °C, which also indicates the presence of carbonaceous compounds. La_2_O_2_CO_3_ is an intermediate phase, stable between 500 and 700 ^◦^C, in the decomposition of La_2_(CO_3_)_3_ and La_2_(OH)_2_(CO_3_)_2_ to La_2_O_3_.^53,58^

## Conclusion

Crystallization of LLZO from a Pechini synthesis-prepared gel has been studied by in situ HTXRD. After calcination at 500 °C, the phases present were La_2_Zr_2_O_7_, Li_2_CO_3_ and La_2_O_2_CO_3_. From the HTXRD data and thermodynamic considerations, a net chemical reaction for the formation of cubic LLZO is proposed. The enthalpy, entropy, and Gibbs energy of this reaction are calculated, and we infer that the amount of excess Li does not influence the reaction to a large degree. However, the sample thickness, and hence the loss of Li, apparently has a significant impact on the reaction and formation of cubic LLZO, and formation was observed at a lower temperature in a thinner sample. With the thinner sample, LLZO decomposed at the highest temperatures, likely due to excessive Li loss. We argue that higher sample surface exposure to the atmosphere enhances initial cubic LLZO formation but also increases Li evaporation, which reduces the availability of lithium for LLZO formation and postpones the completion of the reaction. Excessive Li loss caused decomposition of LLZO into La_2_Zr_2_O_7_ and La_2_O_3_. The configurational entropy of cubic LLZO, due to the disordered Li^+^ sublattice, is important for the stabilization of cubic LLZO at high temperatures.

## Supporting Information Available

DFT-calculated structure parameters for La_2_O_2_CO_3_, thermogravimetry, and mass spectrometry, additional results from Pawley refinements of XRD patterns, and SEM micrographs. A preprint of the manuscript is available at ChemRxiv.^59^ (PDF)
