# Supplementary material for: Phase Evolution and Thermodynamics of Cubic Li6.25Al0.25La3Zr2O12 Studied by High-Temperature X-ray Diffraction
Source: Inorg Chem. 2025 Mar 18;64(12):5856–65. doi: 10.1021/acs.inorgchem.4c03738 (PMC11962839; doi:10.1021/acs.inorgchem.4c03738)
Supplement: Supplementary file 1 — ic4c03738_si_001.pdf [file ic4c03738_si_001.pdf]

Supporting Information: Phase evolution and thermodynamics of cubic $Li_{6.25}Al_{0.25}La_3Zr_2O_{12}$ studied by high-temperature X-ray diffraction

Øystein Gullbrekken,[†] Kristoffer Eggestad,[†] Maria Tsoutsouva,[†,‡] Benjamin A.D. Williamson,[†] Daniel Rettenwander,[†] Mari-Ann Einarsrud,[†] and Sverre M. Selbach[*,†]

†*Department of Materials Science and Engineering, Norwegian University of Science and Technology, NTNU, N-7491 Trondheim, Norway*

‡*Present address: ONERA/DMAS, Chatillon 92320, France*

E-mail: selbach@ntnu.no

Structure parameters for $La_2O_2CO_3$

DFT optimized lattice parameters and atomic positions for the conventional monoclinic (*C2/c*) $La_2O_2CO_3$ structure are displayed in Table S1 and S2, respectively.

Table S1: DFT optimized lattice parameters for the conventional $La_2O_2CO_3$ structure.

a	12.1145 Å
b	6.9956 Å
c	16.3773 Å
α, γ	90°
β	104.265°

Table S2: DFT optimized atomic positions for the conventional $La_2O_2CO_3$ structure.

Specie	a	b	c	Wyckoff
La	0.4481	0.5873	0.0945	8f
La	0.2836	0.9190	0.5942	8f
La	0.6135	0.0815	0.0966	8f
C	0.5000	0.3054	0.2500	4e
C	0.3063	0.7224	0.2502	8f
O	0.8971	0.0817	0.9414	8f
O	0.2852	0.1302	0.2486	8f
O	0.3756	0.7682	0.3211	8f
O	0.4761	0.2165	0.1784	8f
O	0.7701	0.9158	0.0588	8f
O	0.5000	0.5106	0.7500	4e
O	0.3252	0.2343	0.6780	8f
O	0.4334	0.084	0.5525	8f

Thermogravimetry and mass spectrometry

Data from the combined thermogravimetry (TG), mass spectrometry (MS) and differential thermal analysis experiments are provided in Figures S1 and S2.

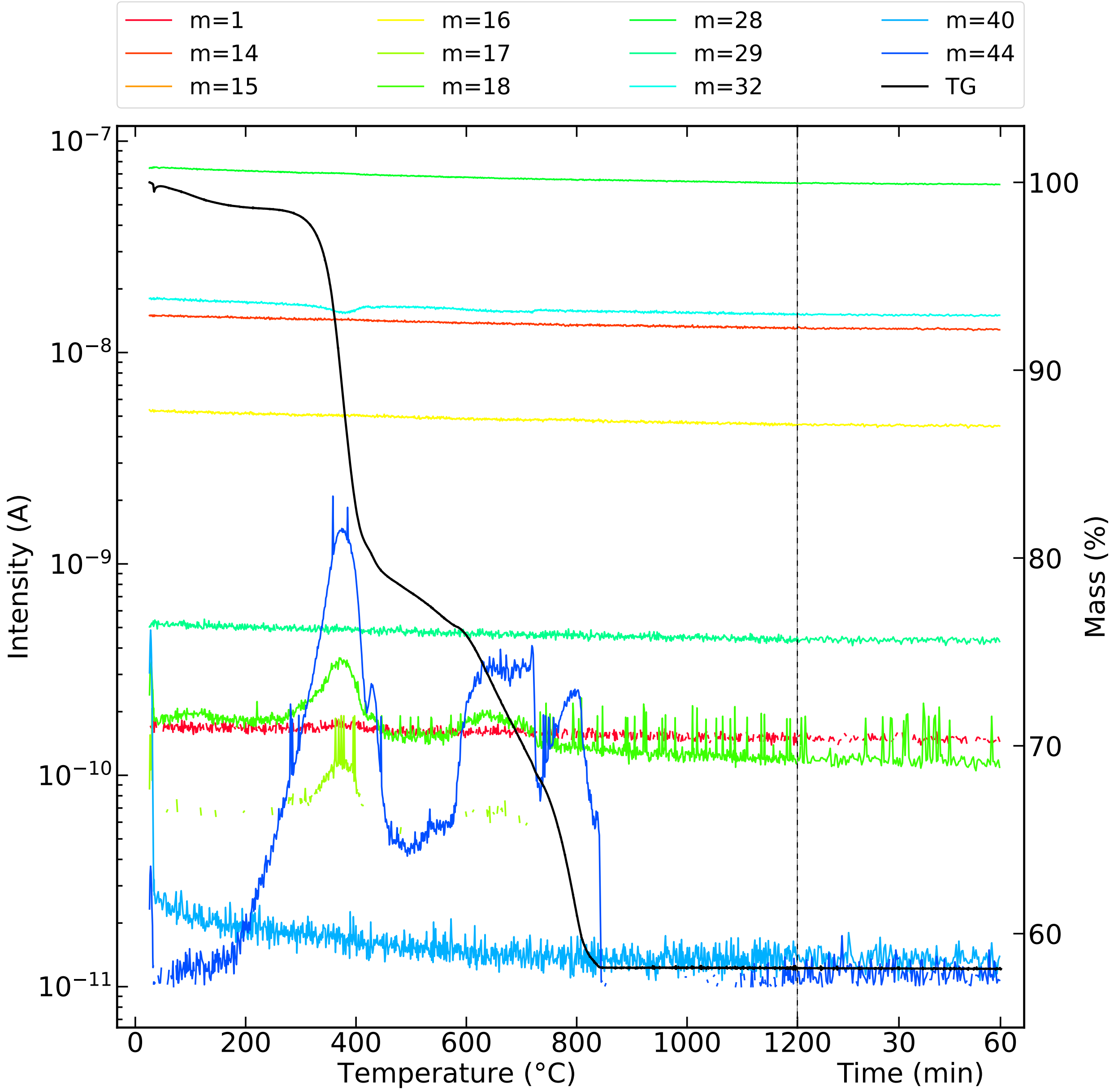

Figure S1: Mass loss (TG) and intensity of mass of species detected by mass spectrometry during TG-MS experiment of precursor powder with 20 % excess Li. Emission of carbon dioxide ($m/z = 44$), water ($m/z = 18$) and OH^- ($m/z = 17$) is visible between 200 and 850 °C from the increase of intensities of the respective mass lines. The data for the precursor powders with 0 and 10 % Li excess were similar.

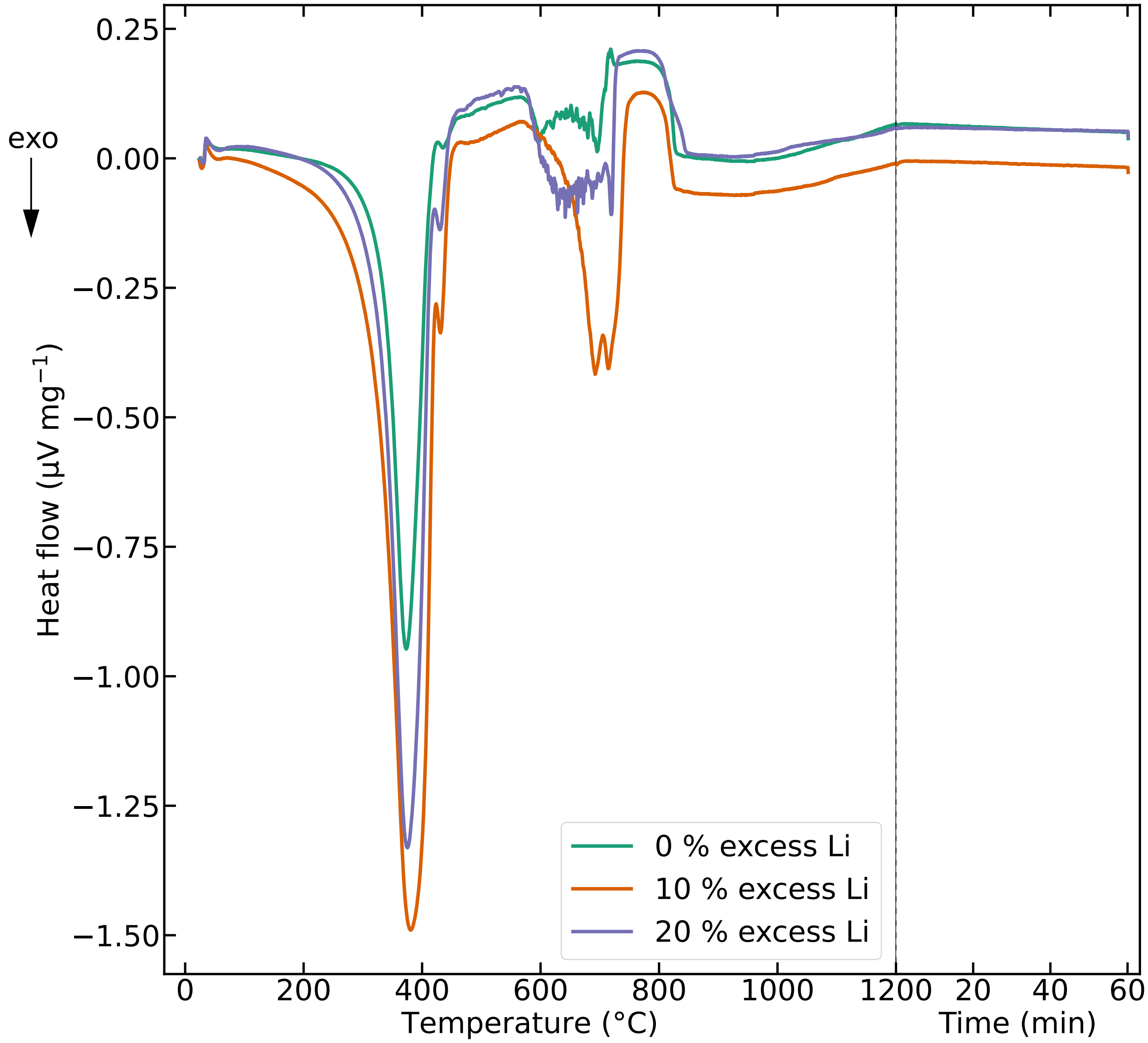

Figure S2: Heat flow measured by differential thermal analysis during TG-MS experiment of precursor powders.

Pawley data

Refined lattice parameters and crystallite sizes of $La_2Zr_2O_7$ as a function of temperature are shown in Figure S3. Note that $La_2Zr_2O_7$ is a minority phase at all temperatures and that refined lattice parameters and crystallite sizes are inherently uncertain because of low intensity reflections, in contrast with LLZO as a majority phase. An example of a Pawley fit to high-temperature X-ray diffraction data of 20 % excess Li powder is displayed in Figure S4.

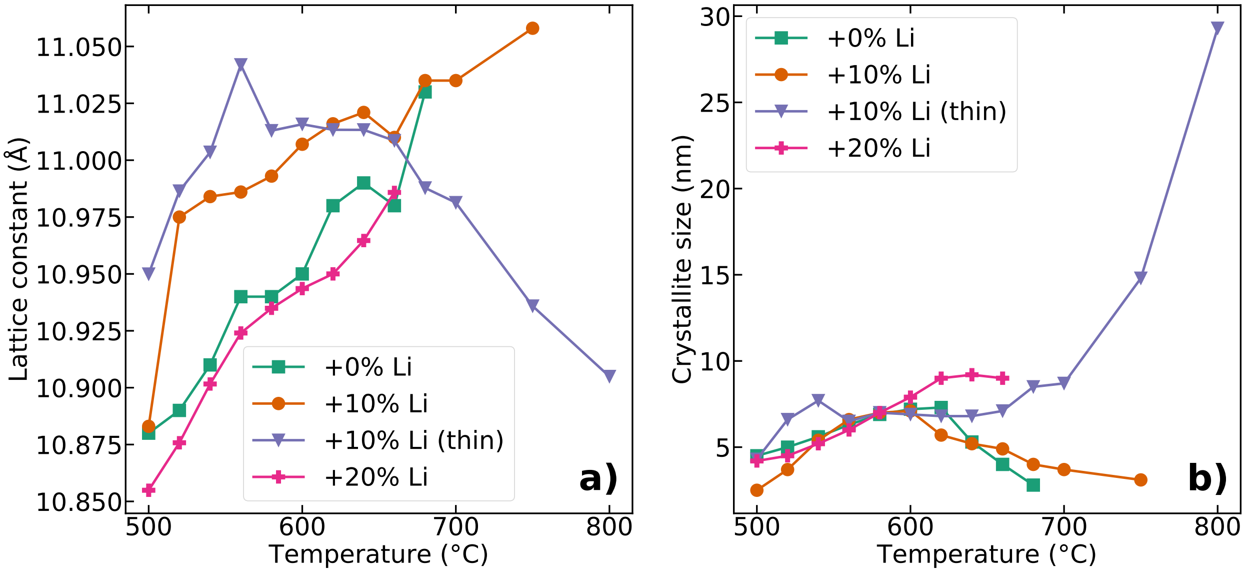

Figure S3: (a) Lattice parameter and (b) crystallite size (LVol-IB) of $La_2Zr_2O_7$ phase during HTXRD experiment.

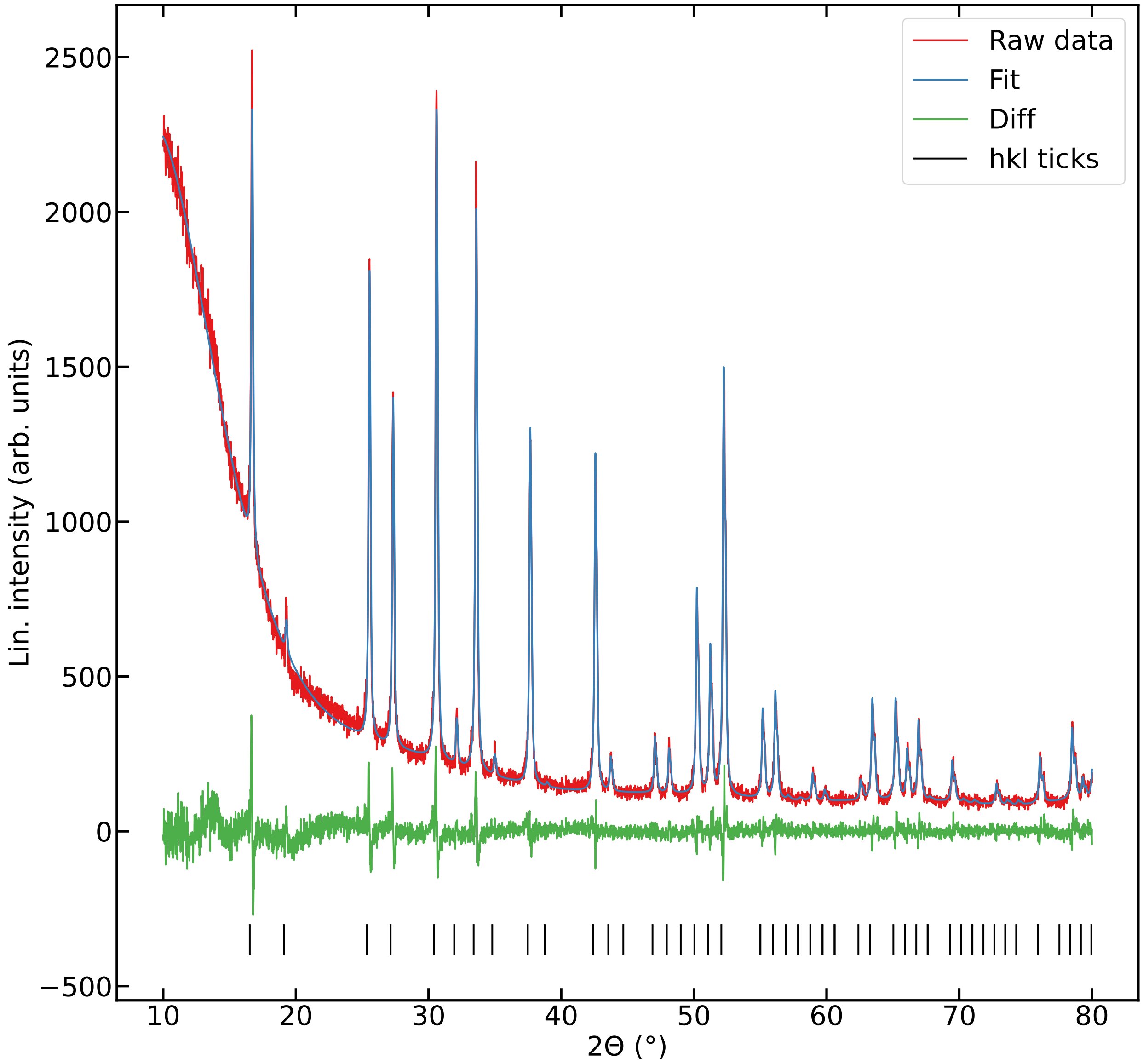

Figure S4: Example of Pawley fit to high-temperature X-ray diffractogram data of LLZO recorded at 800 °C of 20 % excess Li powder.

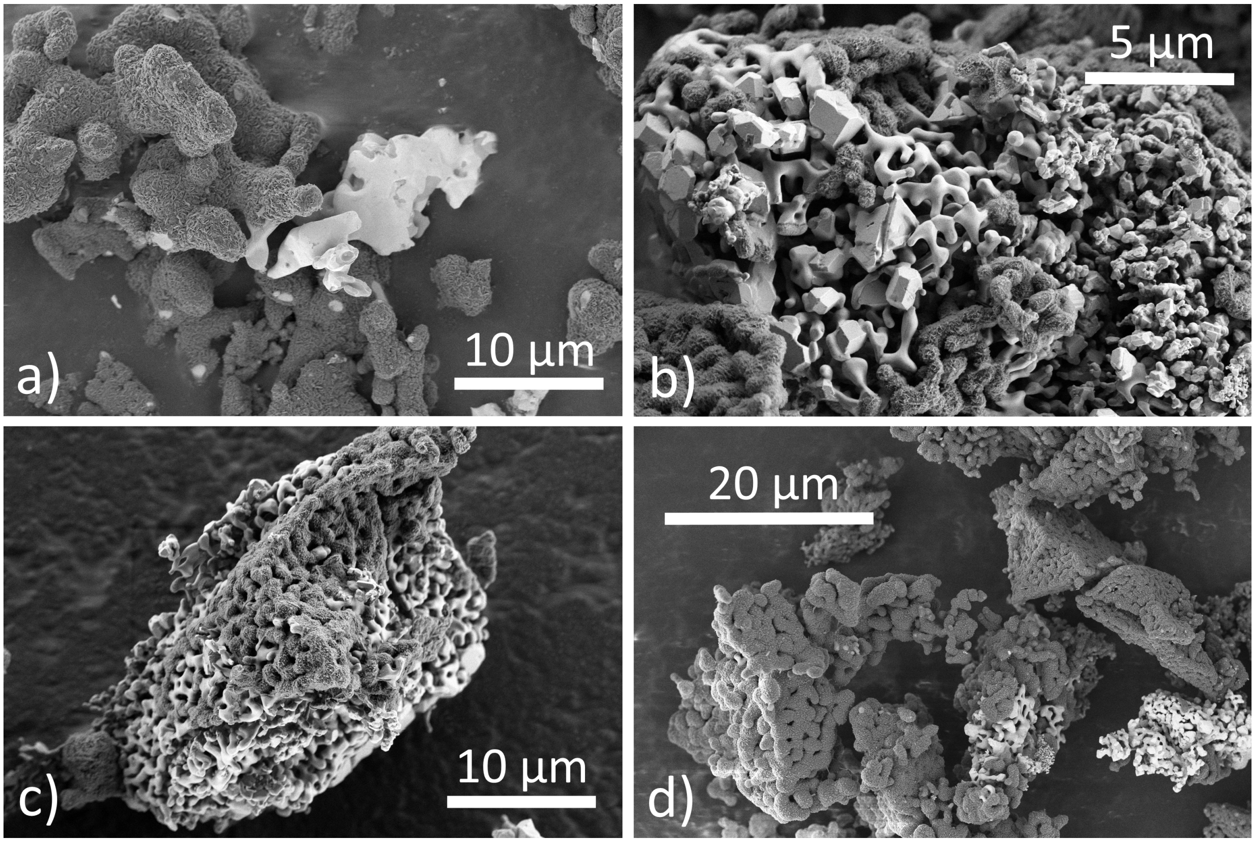

Figure S5: SEM images showing the microstructure of powders after HTXRD experiments. (a) 10 % excess Li, thin deposition. (b) 20 % excess Li. (c) 10 % excess Li, normal deposition. (d) 0 % excess Li.
